# Patients' Costs and Cost-Effectiveness of Tuberculosis Treatment in DOTS and Non-DOTS Facilities in Rio de Janeiro, Brazil

**DOI:** 10.1371/journal.pone.0014014

**Published:** 2010-11-17

**Authors:** Ricardo Steffen, Dick Menzies, Olivia Oxlade, Marcia Pinto, Analia Zuleika de Castro, Paula Monteiro, Anete Trajman

**Affiliations:** 1 Universidade Federal do Rio de Janeiro, Rio de Janeiro, Brazil; 2 Rio de Janeiro TB Scientific League, Rio de Janeiro, Brazil; 3 Montreal Chest Institute, McGill University, Montréal, Canada; 4 Instituto Fernandes Figueira, Fundação Oswaldo Cruz, Rio de Janeiro, Brazil; 5 Universidade Gama Filho, Rio de Janeiro, Brazil; University of Cape Town, South Africa

## Abstract

**Background:**

Costs of tuberculosis diagnosis and treatment may represent a significant burden for the poor and for the health system in resource-poor countries.

**Objectives:**

The aim of this study was to analyze patients' costs of tuberculosis care and to estimate the incremental cost-effectiveness ratio (ICER) of the directly observed treatment (DOT) strategy per completed treatment in Rio de Janeiro, Brazil.

**Methods:**

We interviewed 218 adult patients with bacteriologically confirmed pulmonary tuberculosis. Information on direct (out-of-pocket expenses) and indirect (hours lost) costs, loss in income and costs with extra help were gathered through a questionnaire. Healthcare system additional costs due to supervision of pill-intake were calculated considering staff salaries. Effectiveness was measured by treatment completion rate. The ICER of DOT compared to self-administered therapy (SAT) was calculated.

**Principal Findings:**

DOT increased costs during the treatment phase, while SAT increased costs in the pre-diagnostic phase, for both the patient and the health system. Treatment completion rates were 71% in SAT facilities and 79% in DOT facilities. Costs per completed treatment were US$ 194 for patients and U$ 189 for the health system in SAT facilities, compared to US$ 336 and US$ 726 in DOT facilities. The ICER was US$ 6,616 per completed DOT treatment compared to SAT.

**Conclusions:**

Costs incurred by TB patients are high in Rio de Janeiro, especially for those under DOT. The DOT strategy doubles patients' costs and increases by fourfold the health system costs per completed treatment. The additional costs for DOT may be one of the contributing factors to the completion rates below the targeted 85% recommended by WHO.

## Introduction

The burden of tuberculosis (TB) affects mainly the poor, to whom the costs of accessing TB diagnosis and treatment may represent a significant burden [1]. Brazil is ranked 14^th^ of the twenty-two high burden countries that account for 80 percent of the world's TB burden, with 92,000 new cases each year [2]. Treatment is available free of charge in public healthcare units since the sixties. In Rio de Janeiro, urban violence, poverty, social inequity, and a complex healthcare system contributes to the high incidence of TB, with 75/100,000 new cases reported yearly [3]. To address this problem more effectively, in July of 1999, the Health Secretariat of Rio de Janeiro began to progressively implement the directly observed therapy (DOT) strategy, initially in six of the city's 34 TB clinics [4]. In Rio de Janeiro, the DOT program has been largely clinic-based, with treatment provided in municipal health centers. In clinics where DOT is offered, all patients are treated under supervision. During the intensive phase (first two months), treatment is usually supervised at least three times weekly, ideally five times weekly, followed by the continuation phase (remaining four months), with supervision twice weekly.

In Brazil, as in many other countries, the pathway to TB care is characterized by several and repeated visits to different healthcare providers, which are associated with both system and patient delays [5]–[7]. Although public health services are, in theory, universally available and free of charge for the patients, a substantial portion of the costs still fall on the patients and their families. A thorough understanding of the costs associated with TB diagnosis and treatment is important to develop interventions to reduce that economic burden on patients. Several studies have assessed the patient and household costs of TB and the cost-effectiveness of alternative TB treatment strategies around the world [8]–[13], but these did not consider patient and health system perspectives together. In this study, our primary objective was to analyze the costs of care for tuberculosis patients undergoing treatment in facilities using the DOT and facilities providing only self-administered therapy (SAT) in Rio de Janeiro State (RJ), Brazil. In addition, the extra costs of treatment supervision to the patient and the health system were estimated to calculate the incremental cost-effectiveness ratio (ICER) of the DOT strategy per completed treatment.

## Methods

### Ethics Statement

The study was approved by the ethical committee of Gama Filho University and the Brazilian National Ethical Committee (CONEP, #235/2007). All interviewed patients gave written informed consent.

### Setting

In the RJ, a system for registering cases, a reliable supply of high quality medications with fixed-dose combinations and access to sputum smear microscopy have been in place for decades. Diagnosis and treatment of TB is free of charge. However, DOT has been introduced only recently in some health facilities, with progressive expansion. Supervised treatment is mandatory in clinics where DOT is implemented. Patients on SAT treatment come once monthly for follow up visit and pill collection for the entire month, while patients under DOT come in variable frequencies, depending on the clinics (see extrapolation of costs). Twenty-one health facilities in six TB high burden counties of RJ were selected by convenience (suggested by state and municipal TB control program coordinators), of which 12 used DOT for all patients and 9 offered only SAT.

### Patients

Eligible patients had bacteriologically confirmed pulmonary TB (positive sputum smear and/or culture) with or without concomitant extra-pulmonary involvement, were aged 18 years or older and were being treated at the selected health facility. Patients were recruited from April 2007 to May 2008, after they had completed approximately two months of treatment (from the 5th to the 11th week of treatment). This time point was chosen since it was considered the best compromise between more reliable recall of costs incurred in the pre-diagnostic period while giving the patient sufficient time of treatment to report on costs incurred during treatment. All the patients were approached for the interview while waiting for follow-up visits in the different health centers.

### Overview of the Patient Cost Questionnaire

A standardized questionnaire was used to collect details regarding all costs incurred by the TB patients and their families for each care-seeking episode during the entire period of the patient's TB illness, from the onset of symptoms until the date of interview. Information about direct and indirect costs was gathered. Direct costs were defined as out-of-pocket expenditures, including transportation fees, consultation fees, non-TB laboratory tests, non-TB medication (vitamins, antibiotics, cough syrups and others) and food. TB tests and drugs are offered free of charge in all clinics. Indirect costs were defined as any time lost due to TB illness, including travel time, consultation time, hospitalization, and absenteeism from work. The patient's time and that of his/her family was ascertained. Family time was estimated from the proportion of outpatient visits where a family member escorted the patient, and the proportion of days a family member stayed or visited a patient during his hospitalization.

The number of hours lost were multiplied by the hourly wage in Brazil. The estimated hourly wage was 1.31 American dollars (US$), based on the Brazilian annual minimum wage in 2008, divided by the assumed number of annual hours of work based on a 44 weekly hours of work contract (2,288 hours/year). Help with daily tasks was also registered, whether paid (included as direct costs) or not (included as indirect costs). All out-of-pocket expenditures were recorded using the local currency and converted into US$ considering the exchange rate at the time of the interviews (US$1 = 1.80 *Reais*), based on the currency exchange rates in 2008. Costs are presented according to the period: pre-diagnosis costs were those since the beginning of symptoms to the first confirmatory sputum test and post-diagnosis were those incurred during the treatment phase.

General information regarding the patients' household, employment, and income was also obtained. In addition, patients were asked if their income and their family income decreased after the onset of symptoms, and if the reduction was associated to the illness. The final section of the questionnaire included any additional expenditures or time lost by the patient and their family.

Other important information gathered were the interval from the onset of TB related symptoms until the patient first visited any health facility, including pharmacies or alternative healers (patient delay) and the interval between the patient's first visit to a health facility until the date the patient was officially diagnosed with TB (health system delay).

The frequency and costs related to each medical follow-up and DOT visit were collected separately, since patients typically came monthly for medical follow-up visits, but came three times a week for pill-collection and intake visits. The average time spent and out-of-pocket expenditures were ascertained for the two types of visits from the start of out-patient treatment until the date of interview. These average costs were extrapolated to the projected total number of DOT and follow-up visits throughout the entire duration of the patient's TB treatment, which was assumed to be 6 months, with the frequency of 5 times weekly during the intensive phase and twice weekly during the continuation phase. Direct costs were also ascertained for any extra help received due to illness, including expenses due to help received before or after diagnosis, as well as any extra monthly purchases due to the TB illness.

### Health Facility Costs

Healthcare system additional costs for DOT were calculated based on salary of staff responsible for direct observation of treatment. In RJ, the observation of pill intake is usually done by a nurse or a nurse aid under the supervision of a nurse. In public health facilities, staff with University degrees has equivalent salaries and carrier plans. Since TB diagnosis and treatment had already been available for decades in the municipal health centers and health posts and supervision of treatment is done during the usual opening hours of these healthcare units, with no additional infrastructure needs, we assumed that all costs would be similar in DOT and SAT facilities, except for human resources.

Costs per visit were based on the number of annual patient visits in each facility. The cost of each DOT (pill collection) visit was estimated to be a third of the cost of a patient visit, based on the relative times reported by patients for DOT and medical follow-up visits, and on previous experience [13].

For hospitalization costs and average length of stay, information was gathered in the Ministry of Health online database (available at http://www2.datasus.gov.br/DATASUS/index.php?area=0202). The mean length of hospitalization is 21.9 days, with a reimbursement value of US$ 458.13.

### Cost-effectiveness analysis

The measure of effectiveness was treatment completion rate, since not all patients completing treatment have a bacteriological confirmation of cure. For cost-effectiveness analysis, the incremental cost-effectiveness ratio (ICER) was calculated dividing the costs difference in DOT and SAT facilities by the completion rate difference among interviewed patients under DOT and SAT.

### Data Entry and Statistical Analysis

Data was double-entered into a database created in Microsoft Access 2000 (Microsoft Inc., Virginia, USA). The data was cleaned, any discrepancies were checked against the original questionnaires and the accuracy of data entry was verified. Data analysis was performed using SAS (SAS Institute Inc., North Carolina, USA) by obtaining frequencies, means and standard deviation (SD) or median and interquartile ranges (IQR) when appropriate for patient characteristics and cost variables. For health facilities, average costs and weighted means were calculated. Differences in means where evaluated using T tests, differences in medians were evaluated using the Mann–Whitney U test and difference in proportions were analyzed using the *chi*-square test. Costs were extrapolated for the total number of new cases registered yearly in Rio de Janeiro state (15,000).

### Sensitivity Analyses

Sensitivity analyses were performed to explore the degree of uncertainty of the treatment outcomes, the costs of follow-up and pill-collection visits, the costs of hospitalization and the frequency of weekly pill-collection visits. The total staff cost values varied from 25% to twice the costs of the base case. The frequencies of pill-collection visits ranged from the most frequently observed of 60 (corresponding to three visits weekly during the intensive phase and twice weekly visits during the continuation phase) to 120 (five weekly visits during the entire treatment) total visits. Hospitalization costs varied with the length of stay and with the difference in hospitalization rates.

## Results

### Overall patient costs

A total of 218 patients were interviewed, 103 (47%) under SAT and 115 (53%) under DOT. Their characteristics were similar, except for time to reach the healthcare unit from home, which was longer for SAT patients, as displayed in Table 1. Mean cost per patient was US$ 336 (SD = 540) for patients under DOT and US$ 194 (SD = 311), for patients under SAT, p = <0.01 (Table 2). Considering an incidence of 15,000 new cases per year in Rio de Janeiro, the DOT strategy accounted for an extra US$ 2,135,000 (95% CI = US$ 1,245,000-US$ 3,000,000) annual cost for patients and their families when compared to the SAT, out of which 50% was from indirect (lost wages or lost opportunity) costs.

**Table 1:** Characteristics of 218 patients interviewed in Rio de Janeiro state, Brazil, according to the treatment strategy.

	DOT *N (%)*	SAT *N (%)*	*P* value
**Sex**			
Female	42 (36.5)	37 (35.9)	0.93
Male	73 (63.5)	66 (64.1)	
**Age**			
<35	55 (47.8)	59 (57.3)	0.45
≥35	60 (52.2)	44 (42.7)	
**Schooling**			
None	16 (13.9)	13 (12.6)	0.16
Primary	57 (49.6)	46 (44.6)	
Unfinished High School	6 (5.2)	15 (14.6)	
Finished High School	31 (27.0)	22 (21.4)	
University	5 (4.3)	7 (6.8)	
**Household size**			
Lives alone	13 (11.3)	12 (11.7)	0.98
2 to 4	75 (65.2)	68 (66.0)	
>5	27 (23.5)	23 (22.3)	
Monthly income (US$)*			
< $55	68 (59.1)	64 (62.1)	0.68
55–222	27 (23.5)	24 (23.3)	
223–444	7 (6.1)	8 (7.8)	
>444	13 (11.3)	7 (6.8)	
Household monthly income (US$)*			
< $55	29 (25.2)	28 (27.2)	0.27
55–222	30 (26.1)	35 (34.0)	
223–444	21 (18.3)	20 (19.4)	
>444	35 (30.4)	19 (19.4)	
**Employment status**			
Employed	60 (52.2)	58 (56.3)	0.42
Student	9 (7.8)	3 (2.9)	
Unemployed	38 (33.0)	33 (32.1)	
Retired	8 (7.0)	9 (8.7)	
**Co-morbidities**			
No	75 (65.2)	70 (67.0)	0.66
Yes	40 (34.8)	33 (32.0)	
**Type of first searched facility**			
Public Primary Care Unit	31 (27.0)	39 (37.9)	0.29
Pharmacy	12 (10.4)	9 (8.7)	
Hospital	64 (55.6)	44 (42.7)	
Private Clinic	7 (6.1)	10 (9.7)	
Others	1 (0.9)	1 (1.0)	
**Time to reach healthcare unit**			
0–20 min	44 (38.3)	19 (18.4)	**<0.01**
21–40 min	28 (24.4)	21 (20.4)	
>40 min	43 (37.4)	63 (61.2)	
**Health Insurance**			
No	94 (81.7)	90 (87.4)	0.25
Yes	21 (18.3)	12 (16.2)	
**Hospitalization**			
No	97 (84.4)	92 (89.3)	0.28
Yes	18 (15.6)	11 (10.7)	
**History of previous TB treatment**			
New case	95 (82.6)	92 (89.3)	0.16
Retreatment	20 (17.4)	11 (10.7)	

N =  number of patients in each category.

SAT = self-administered therapy.

DOT = directly observed therapy.

**Table 2:** Direct and indirect costs (in US$) incurred by 218 patients before and after diagnosis according to the treatment strategy.

	DOT (N = 115)		SAT (N = 103)		
	*n*	Mean cost (SD)	*n*	Mean cost (SD)	*P value*
**Delay in diagnosis (in days)**					
Patient delay	-	64.3 (114.4) days	-	88.9 (219.3) days	0.31
System delay	-	36.4 (56.0) days	*-*	52.5 (105.3) days	0.17
**Before diagnosis**					
Direct					
Transport	*92*	11.0 (14.7)	*82*	12.7 (14.9)	0.42
Consultation fees	*10*	2.2 (10.1)	*16*	7.1 (23.2)	**0.05**
Complementary exams	*11*	3.1 (10.3)	*27*	6.9 (19.3)	0.08
Non-TB medication	*48*	10.7 (22.7)	*44*	16.5 (30.4)	0.11
Food	*43*	2.1 (3.4)	*50*	3.1 (7.2)	0.16
*Sub-total*	*102*	*29.1 (37.8)*	*103*	*46.3 (61.2)*	0.11
Indirect					
Value of time on trips to clinic and absenteeism	*115*	39.5 (180.6)	*101*	30.1 (132.4)	0.37
Value of family time escorting patient and absenteeism	*64*	5.6 (21.8)	*72*	3.1 (4.8)	0.4
*Sub-total*	*115*	*45.1 (183.6)*	*103*	*33.2 (132.5)*	0.11
*Total before diagnosis*	*115*	*74.2 (187.5)*	*103*	*79.5 (146)*	0.38
**After diagnosis**					
Direct					
Pill collection/follow-up visits	*115*	115.9 (371)	*103*	37.3 (55.2)	**0.05**
Indirect					
Value of time for clinic visits and absenteeism	*109*	76.9 (141.4)	*100*	33.9 (33.8)	**<0.01**
Value of family time lost for clinic visits and absenteeism	*74*	37.1 (188.4)	*57*	16.7 (31.9)	0.03
*Sub-total*	115	*114 (326.7)*	*103*	*50.6 (57.4)*	
*Total after diagnosis*	*115*	*229.9 (494.3)*	*103*	*87.9 (79.6)*	<0.01
**Hospitalization costs**					
Direct	*18*	5.6 (30.2)	*11*	15.0 (131.7)	0.48
Indirect	*18*	26.7 (104.3)	*11*	11.6 (48.9)	0.08
**Total patient costs**					
Total direct	*108*	150.6 (374.1)	*103*	98.6 (155.4)	0.78
Total indirect	*115*	185.8 (389)	*103*	95.4 (124.4)	**<0.01**
**Total cost**	***115***	**336.4 (539.7)**	***103***	**194 (199)**	**<0.01**

SAT = self-administered therapy; DOT = directly observed therapy. N = Number of people in each category; *n* =  Number of people who reported any cost (mean values include those patients who reported costs zero). US$1.00 = R$1.80.

Hospitalization and female gender were associated with higher costs, regardless of the type of treatment strategy. Twenty-nine (13.3%) patients were hospitalized at some point for a median time of 7 days (IQR = 2–20) and a mean time of 20.8 days. Patients who were hospitalized had a mean cost of US$ 426 (SD 622) as compared to US$ 235 (SD 398, p<0.01) for those who were not hospitalized. Women had a mean cost of US$ 354 (SD 630) versus US$ 214 (SD 269) for male, p = 0.02.

Expenditures resulted mainly from transportation fees and from medication for patients under DOT as well as those under SAT (Table 2). DOT increased costs during the treatment phase, due to increased indirect costs, while patients under SAT had higher costs in the pre-diagnostic phase. Although not statistically significant, total time to diagnosis was 40% longer in SAT facilities (Table 2). For patients under DOT, factors associated with higher costs were living alone, unemployment and hospitalization (data not shown). For those under SAT, factors associated with higher costs were older age, co-morbidities, hospitalization and history of previous treatment (data not shown).

Approximately 35% of patients reported that their income decreased, most (75%) because of TB. Extra help was needed by 102 (47%) patients, who incurred a total of US$ 8,821, exclusively for indirect costs of time spent by family or friends helping these patients.

### Overall health system costs

The average health system cost per follow up visit was estimated to be US$ 22.7 in DOT facilities and US$ 22.2 in SAT facilities. The cost per DOT/pill-collecting visit was estimated to be US$ 7.56. Extrapolating for a six-month TB treatment regimen, the total health system cost per patient for the DOT strategy was US$ 726 and US$ 189 for the SAT strategy.

### Treatment outcomes

Among the 218 interviewed patients, 164 (75%) completed treatment, 46 (21%) defaulted, 7 (3%) failed and 1 died (0.5%). Outcomes according to treatment strategy are displayed in Tables 3 and 4. Completion rates were higher in DOT facilities, although this was not statistically significant [79% (72%;86%) versus 71% (62%;80%), p = 0.21].

**Table 3:** Patient costs according to outcome in DOT facilities.

Outcome	%	Patient delay (days)	System delay (days)	Pre-diagnosis period (US$)	Hospitalization (US$)	Post-diagnosis period (US$/month)	Total (US$)
Completed (n = 91)	79.1%	51.6 (74.2)	36.1 (55.9)	111.4 (515.1)	153.4 (526.8)	36.8 (38.5)	485.5 (764.3)
Default (n = 20)	13.4%	126.8 (217.2)	31.8 (44.8)	48.7 (47.7	279.6 (794)	32.6 (20.5)	523.7 (745.4)
Failure (n = 4)	3.5%	39.4 (43.0)	66.8 (106.8)	16.8 (14.6)	0 (0)	25.9 (10.4)	172.4 (67.5)
Death (n = 0)	0 (0%)	-	-	-	-	-	-

US$1.00 = R$1.80.

**Table 4:** Patient costs according to outcome in non-DOT facilities.

Outcome	%	Patient delay (days)	System delay (days)	Pre-diagnosis period (US$)	Hospitalization (US$)	Post-diagnosis period (US$/month)	Total (US$)
Completed (n = 73)	70.9%	94.5 (248.9)	41.2 (50.3)	72.8 (86.8)	82.5 (349.9)	14.8 (9.6)	244.1 (374.4)
Default (n = 26)	25.2%	70.0 (127.9)	59.1 (172.3)	52.3 (63.2)	68.1 (227.5)	11.8 (11.2)	191.2 (257.8)
Failure (n = 3)	2.9%	27.5 (28.4)	58.0 (8.5)	82.5 (62.9)	0 (0)	7.9 (3.1)	129.7 (81.3))
Death (n = 1)	1 (1%)	225.0 (190.9)	364.5 (51.6)	241.3 (327.8)	0 (0)	19.3 (19.1)	357.1 (443.7)

US$1.00 = R$1.80.

### Costs per case completing treatment

**Figure 1:**
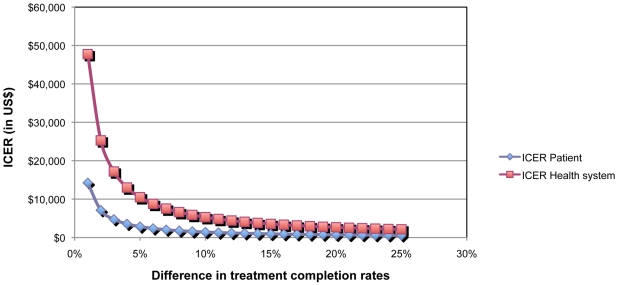
Incremental Cost-Effectiveness Ratio (ICER) according to different treatment completion rates in DOT facilities. The incremental cost-effectiveness ratio was calculated according to an increasing treatment completion rate in treatment facilities offering DOT. The red line represents the ICER taking into account only the costs to the health system.

The average cost-effectiveness ratio for the SAT strategy was calculated to be US$ 266 per completed treatment and US$ 919 per completed treatment for the DOT strategy. From the health system perspective, the ICER of the DOT strategy compared to the SAT strategy was US$ 6,616 per patient completing treatment. From the patient perspective, the ICER was US$ 1,780 per patient completing treatment. Varying the difference in treatment completion rates from 1% to 25%, the incremental cost-effectiveness ratio increased from US$ 3,554 to US$ 47,744 from the health system perspective and from US$ 742 to U$ 11,140, from the patients' perspective (Figure 1 and Table 5). Other sensitivity analysis results are displayed in Table 5.

**Table 5:** Sensitivity analyses for varying differences of outcomes, length and rates of hospitalization, staff salaries and frequency of treatment supervision.

	Health system perspective ICER (US$/patient completing treatment)	Patient's perspective ICER (US$/patient completing treatment)
**Percentage of difference in treatment completion rates**		
Minimum (1%)	US$ 47,744/pct	US$ 14,240/pct
*Base case (8%)*	*US$ 6,616/pct*	*US$ 1,780/pct*
Maximum (25%)	US$ 3,554/pct	US$ 949/pct
**Length of hospitalization (days)**		
−25%	-	US$ 1,762/pct
*Base case (21 days)*	*-*	*US$ 1,780/pct*
25%	-	US$ 1,869/pct
50%	-	US$ 1,886/pct
100%	-	US$ 1,923/pct
200%	-	US$ 1,994/pct
**Percentage of difference in hospitalization rates**		
−5%	US$ 6,899/pct	-
*Base case 0%*	*US$ 6,616/pct*	-
5%	US$ 6,335/pct	-
10%	US$ 6,053/pct	-
15%	US$ 5,771/pct	-
**Staff salary**		
−75%	US$ 1,654/pct	-
−50%	US$ 3,308/pct	-
−25%	US$ 4,965/pct	-
*Base case*	*US$ 6,616/pct*	-
100%	US$ 15,555/pct	-
**Total number of pill-collection visits**		
60 visits (3 weekly visits x 2 mo +2 weekly visits x 4 mo)	US$ 5,313/pct	US$ 650/pct
*72 visits (3 weekly visits x 6 mo) – base case*	*US$ 6,616/pct*	*US$ 1,780/pct*
88 visits (5 weekly visits x 2 mo and 3 weekly visits x 4 mo)	US$ 10,528/pct	US$ 2,728/pct
120 visits (5 weekly visits for 6 mo)	US$ 12,390/pct	US$ 3,474/pct

ICER = Incremental Cost-Effectiveness Ratio.

pct = patient completing treatment.

US$1.00 = R$1.80.

## Discussion

In the present study, a high financial burden was identified among patients with pulmonary TB. One third of patients reported a reduction in income, mostly due to TB disease. Lost hours of work for diagnosis and supervision of treatment were the main source of costs. Hospitalization was associated with a significant increase in costs, a finding already reported in other countries [13]. Efforts for an earlier diagnosis and prevention of complicated and resistant forms of TB would likely reduce costs of the disease by reducing the need for hospitalization.

Unlike previous findings in other countries [13], women incurred in higher costs. Other patients' socio-demographic characteristics were irrelevant for costs. Higher costs related to family income and level of instruction may have been missed because the hourly wage was calculated based on minimum wage rather than on declared family income. On the other hand, costs may have been overestimated because indirect costs were taken into account even for unemployed persons.

DOT was associated with the highest increase in costs, mostly from time lost for collection/observation of pill intake, despite the longer travel time to achieve the health facility taken by patients treated under the SAT strategy. Mohan et al. [14] used a different method to value the patients' indirect costs for pill-collection visits and found that they doubled. Using a more detailed approach, we confirmed these data, but we also captured substantial increase of direct costs.

Treatment completion rates were higher for patients under DOT, although this was not statistically significant. The effectiveness of DOT is consistent with the official RJ programmatic data [14], [15]. However, the overall effectiveness of TB treatment in RJ is 61%, but 19% of outcomes are unknown, thus we believe our data to be a more reliable representation of the overall effectiveness. The additional cost of DOT for patients and their families is substantial, and may be contributing, in Rio de Janeiro, to the treatment completion rates below the targeted 85% recommended by WHO. Despite a reported increase in effectiveness in some settings [14], [16], [17] in poor-resource countries, DOT may be unaffordable, at least for the poorest.

Because of the modest increase in the treatment completion rate with DOT, the ICER was high: US$ 6,616 for the health system and US$ 1,780 for the patient. Sensitivity analyses showed that in varying scenarios, ICER for patients would remain high. The variable with the highest impact in ICER for patients was the total number of pill collection visits, corresponding to a frequency not considered as DOT by the WHO. Conversely, the variable with the smallest impact in the ICER for patients was different lengths of hospitalization. This is a reflection of the small percentage of hospitalization (13%) yielding a modest average indirect cost due to hospitalization. Oddly, the present sample had a higher rate of hospitalization for DOT patients. Nevertheless, we performed a sensitivity analysis assuming that it would be lower. The variation in the ICER was small because the total amount of reimbursement for hospitalization of each TB patient is undervalued.

The present study has a few limitations. First, economic assessment in health is only one of the many tools for decision making. Equity, ethics, resource availability and willingness to pay, patients' preferences and quality of life are other important aspects to be considered. In addition, the current analysis does not take into account the secondary benefits of treating one patient, such as diminished transmission, effects on MDR prevalence and number of deaths, thus underestimating the costs of SAT. Conversely, opportunity costs regarding human resources allocated for treatment supervision were not accounted for, thus underestimating DOT costs. In addition, we did not estimate the costs associated with the implementation of DOT, such as training, previously reported in the city of Rio de Janeiro [14]. Finally, we interviewed patients on their 2^nd^ month of treatment, those who defaulted before the interview were not captured in the analysis. This may have hampered the effectiveness analysis.

Despite these limitations, this study clearly shows that a significant proportion of the additional costs for DOT fall on the patient, and that must be acknowledged to achieve a more effective TB control strategy.

## Acknowledgments

The authors are thankful for all students of Rio de Janeiro TB Scientific League who helped with data collection.
